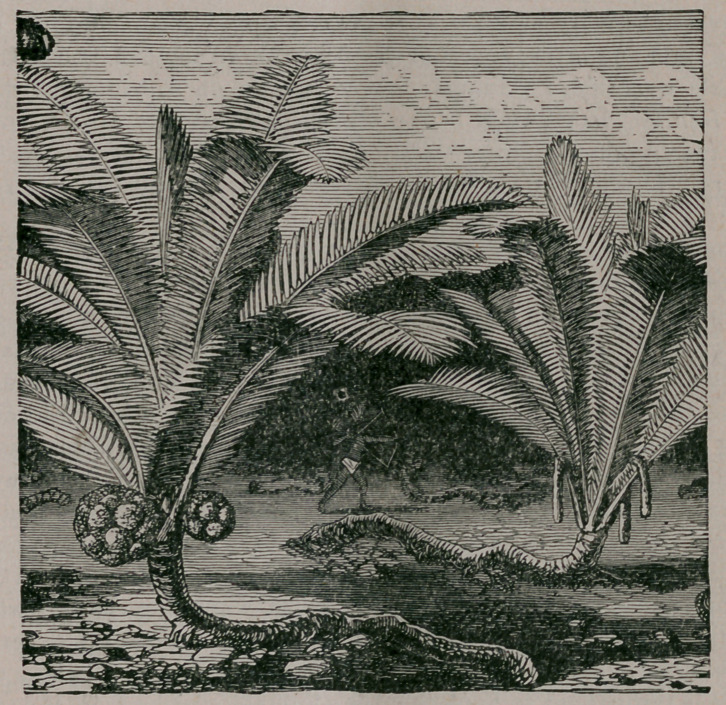# The Ivory Plant

**Published:** 1889-06

**Authors:** 


### THE IVORY PLANT.

So different are the products of the animal from those of the vegetable kingdom, that even the most careless observer may be expected at once to distinguish them. Yet multitudes are in the daily use of ivory buttons, boxes and small ornaments, who never doubt that they are made from the tusks of the elephant, while they are really the product of a plant. The ivory plant is a native of the northern regions of South America, extending northward just across the Isthmus of Panama, large groves of it have been recently discovered in the province of that name.

It is found in extensive groves—in which it banishes all other vegetation from the soil it has taken possession of—or scattered among the large trees of the virgin forests. It has the appearance of a stemless palm, and consists of a graceful crown of leaves, twentv feet long, of a delicate pale green color, and divided like the plume of a feather into from thirty to fifty pairs of long narrow leaflets. It is not, however, really stemless, but the weight of the foliage and the fruit is too much for the comparatively slender trunk, and consequently pulls it down to the ground, where it is seen like a large exposed root, stretching for a length of nearly twenty feet in the old plants. The long leaves are employed by the Indians to cover the roofs of their cottages.

Each flower of the ivory plant does not contain stamens and pistils, as in most of the British plants, but like our willows, one tree produces only staminal flowers, while another has only pistillate ones. Such plants are said by botanists to be diœcious. Both kinds of the plants of the vegetable ivory have the same general appearance, and differ only in the form and arrangement of the flowers. In the one kind an innumerable quantity of staminal flowers is born on a cylindrical fleshy axis, four feet long, while in the other a few pistillate flowers spring from the end of the flower-stalk. Each plant bears several heads of flowers. Purdie, who visited the plants in their native locality in 1846, says :

The fragrance of the flowers is most powerful, and delicious beyond that of any other plant, and so diffuse, that the air for many yards around was alive with myriads of annoying insects, which first attracted my notice. I had afterwards to carry the flowers in my hands for twelve miles, and though I killed a number of insects that followed me, the next day a great many still hovered about them, which had come along with us from the wood where the plant grew. The group of pistillate flowers produce a large roundish fruit, from eight to twelve inches in diameter, and weighing when ripe about twenty-five pounds. It is covered by a hard woody coat, everywhere embossed with conical angular tubercles, and is composed of six or seven portions, each containing from six to nine seeds. These seeds, when ripe, are pure white, free from veins, dots, or vessels of any kind, presenting a perfect uniformity of texture surpassing the finest animal ivory ; and its substance is throughout so hard that the slightest streaks from the turning-lathe are observable. Indeed, it looks much more like an animal than a vegetable product ; but a close comparison will enable one to distinguish it from the ivory of the elephant by its brightness and its fatty appearance, but chiefly by its minute cellular structure. This curious hard material is the store of food laid up by the plant for the nourishment of the embryo, or young plant contained in the seed. It corresponds to the white of an egg of the hen, and has been consequently called the albumen of the seed. In its early condition this ivory exists as a clear insipid fluid, with which travelers allay their thirst ; afterwards the liquor becomes sweet and milky, and in this state it is greedily devoured by bears, hogs and turkeys ; it then gradually becomes hard. It is very curious that this hard mass again returns to its former soft state in the process of germination. The young plant for some time is dependent upon it for its food, and if the seed be taken out of the ground after the plant has appeared, it will be found to be filled with a substance half pulp and half milk, on which the plant lives until it is old enough to obtain its food on its own account.

From the small size of the seed, the largest not being more than two inches across its greatest diameter, the vegetable ivory can be employed in the manufacture of only small articles, such as beads, buttons, toys, etc. What is wanting in size is, however, often made up by the skill and ingenuity of the workmen, who join together several pieces so as to make a long object, when it is easy to hide the joints from view, or make a lid from one seed, and the box from another.

## Figures and Tables

**Figure f1:**